# Antifungal and Antibiofilm Activities of 2-Aminobenzoic Acid Derivatives Against a Clinical Ocular *Candida albicans* Isolate for Biomedical Applications

**DOI:** 10.3390/antibiotics14050432

**Published:** 2025-04-25

**Authors:** Francesco Petrillo, Angela Maione, Marisa Spampinato, Lea Di Massa, Marco Guida, Armando Zarrelli, Emilia Galdiero, Luigi Longobardo

**Affiliations:** 1 Department of Medical Sciences, Eye Clinic, Turin University, 10024 Turin, Italy; fpetrillo@cittadellasalute.to.it; 2 Department of Biology, University of Naples ‘Federico II’, Via Cinthia, 80126 Naples, Italy; angela.maione@unina.it (A.M.); marisa.spampinato@unina.it (M.S.); le.dimassa9@gmail.com (L.D.M.); marco.guida@unina.it (M.G.); 3 BAT Center—Interuniversity Center for Studies on Bioinspired Agro-Environmental Technology, University of Naples Federico II, 80055 Portici, Italy; 4 Department of Chemical Science, University of Napoli Federico II, Via Cinthia 4, 80126 Naples, Italy; zarrelli@unina.it (A.Z.); luilongo@unina.it (L.L.)

**Keywords:** antibiofilm, antimicrobials, *Candida albicans*, 2-aminobenzoic acid derivatives, gene expression

## Abstract

Ocular fungal infections are slow-progressing conditions that primarily affect the cornea but can also involve the entire eyeball. *Candida albicans* is one of the most involved species. Both diagnosing and treating these infections require prompt and effective action. However, the currently available treatment options mainly rely on azoles and polyenes, which are known for their poor penetration into ocular tissue and associated toxicity. Moreover, conventional antifungals are usually ineffective when tested against biofilm-associated infections, mainly due to the metabolically inactive state of dormant cells embedded in the extracellular biofilm matrix. Here, analysis of the *in vitro* antifungal activity of four 2-aminobenzoic acid derivatives synthesized using a green method and their combination with Fluconazole (FLC) showed efficacy against the FLC-resistant clinical isolate of *C. albicans* under both planktonic and biofilm formation conditions. Results showed that compounds **1** and **2** exhibited the best antifungal activity in the checkerboard association test, presenting a synergistic effect towards antifungal action. The downregulation of *HWP*, *ERG11*, and *ASL3* genes during biofilm inhibition suggested a reduced capacity of the four compounds for hyphal growth and adhesion, as well as a decrease in pathogenicity due to the downregulation of some *SAP* genes. *In vitro* and *in vivo* toxicity profiles indicated that these compounds exhibited low toxicity, as well as the absence of genotoxic effects. Therefore, green-synthetized 2-aminobenzoic acid derivatives may have potential as antifungal agents for the inhibition of *C. albicans* growth and biofilm formation.

## 1. Introduction

Fungal infections represent an increasingly serious threat to human health, creating a great clinical challenge due to the limited availability of effective antifungal treatments. This leads to a lack of treatment options when infections develop resistance to existing drugs. Recently, the World Health Organization (WHO) introduced its first fungal priority pathogen list, which emphasizes the serious impact of fungal diseases on human health and identifies antifungal resistance as a “priority” [1]. Fungal infections are a significant contributor to microbial diseases of the eye and are particularly prominent in tropical and low-income regions, where they rank among the leading causes of vision loss. These infections can impact various parts of the eye, including the eyelids, conjunctiva, lacrimal system, sclera, cornea, uveal tract, and deeper intraocular areas. Severe infections that threaten vision include keratitis, an inflammation of the cornea that may result in scarring and loss of clarity, and endophthalmitis, a serious intraocular infection that can rapidly lead to permanent blindness. Among the fungal pathogens affecting the eye, *Candida* species are especially noteworthy for their clinical relevance [1]. *Candida* species are recognized as medically significant yeasts due to their high prevalence in colonizing and infecting the human host. The *Candida* genus includes over 150 species, some of which are part of the normal microbiota in healthy individuals. However, under specific conditions, such as in immunocompromised patients, these yeasts can shift from a commensal state to pathogenic microorganisms, becoming opportunistic pathogens. *Candida* infections can vary from superficial skin and mucosal lesions to severe, life-threatening systemic diseases. *Candida albicans* is the most isolated species from various body sites [2]. Several virulence factors contribute to the pathogenicity of *C. albicans*, including morphogenesis and biofilm formation. Morphogenesis plays a significant role in its virulence, allowing *C. albicans* to penetrate mucosal membranes, invade tissues, and enter the bloodstream, increasing the potential for tissue damage and infection. This process ultimately leads to the formation of biofilms, another key virulence factor. Biofilms consist of yeast and pseudo-hyphal and hyphal cells embedded in a polymeric extracellular matrix (EPS). Planktonic cells play a key role in the early stages of biofilm formation by attaching to surfaces, where they grow as individual yeast cells or hyphal filaments. Additionally, after biofilm maturation, an active phase called dispersion occurs, during which fungal cells detach from the biofilm and return to their planktonic form. This detachment can lead to the spread of cells to other sites, initiating a new cycle of biofilm formation. In *C. albicans*, this process contributes to candidemia and the spread of infection, leading to invasive disease [3,4]. Within biofilms, the cells are shielded from immune responses and antifungal treatments. Consequently, the pathogenicity of *C. albicans* is primarily driven by biofilm formation, with a key morphological feature being the transition from the yeast form to the hyphal form. Currently, three main classes of antifungal drugs are used to treat severe *C. albicans* infections: azoles, echinocandins, and polyenes. However, many of these drugs are toxic and have a limited spectrum of activity. Moreover, their prolonged use can lead to resistance in previously susceptible *Candida* strains [5]. The range of commercially available antifungal agents is limited in comparison to the extensive array of antibacterial drugs. Additionally, the development of new antifungal agents that are both effective and non-toxic to the human host is challenging, as fungal cells are also eukaryotic. Given the limited availability of antifungals, their potential adverse effects, and the rise of antifungal resistance, there is an ongoing need to discover new molecules that target alternative fungal cell mechanisms while minimizing toxicity to mammalian hosts [6,7].

**Figure 1:**
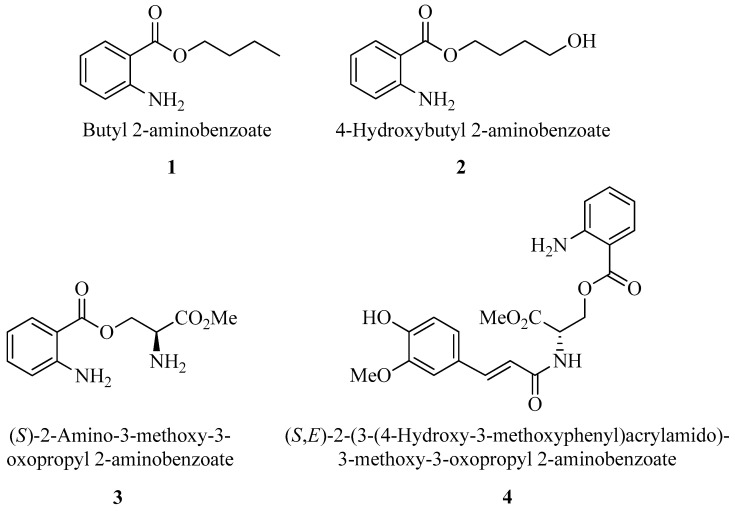
Derivatives of 2-aminobenzoic acid utilized in this work.

2-Aminobenzoic acid (also known as anthranilic acid) derivatives are notable for their versatility in medicinal chemistry, as they are key intermediates in synthesizing various drugs, like non-steroidal anti-inflammatory drugs, diuretics, and anti-allergic agents. These compounds are also recognized for their potential anticancer, antimicrobial, and antiviral properties, which may offer therapeutic benefits against a range of diseases [8]. Previous investigations focusing on quantitative structure–activity relationships and in vitro assays have indicated that specific derivatives of 2-aminobenzoic acid, particularly those with the carboxyl esterified with simple alkyl groups and possessing a free amino group, have an increased efficacy against bacterial and fungicidal infections, particularly those caused by *Staphylococcus aureus*, *Bacillus subtilis*, *Escherichia coli*, *Candida albicans*, and *Aspergillus niger* [9]. Here, we considered some simple esters of 2-aminobenzoic acid and some more complex ones containing amino acid serine and its conjugate with ferulic acid. The first three ester derivatives (**1**–**3**) were prepared using a green synthetic procedure that avoids toxic reagents like SOCl_2_, which have been used in previous studies [9]. The last one (**4**), incorporating hydroxycinnamic acids, was obtained by conjugating serine methyl ester with ferulic acid through an amide bond, employing a novel sustainable protocol for the chemical derivatization of hydroxycinnamic acids [10]. The structures of the molecules prepared and tested in this work are illustrated in Figure 1. Additionally, we investigated the antifungal potential of these compounds against *C. albicans* in planktonic and biofilm forms, as well as their synergistic potential with common antifungal agent Fluconazole (FLC). Their *in vitro* and *in vivo* toxicity was also investigated using human keratinocyte cell line HaCAT and the nematodes model to find new inhibitor candidates.

## 2. Results

### 2.1. Design and Synthesis of Derivatives of 2-Aminobenzoic Acid

**Scheme 1:**
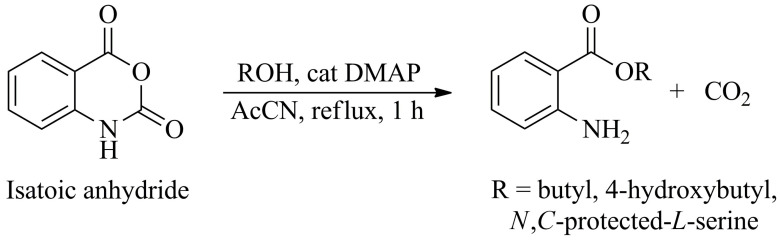
Synthesis of alkyl 2-aminobenzoate.

The assembly of molecules containing 2-aminobenzoyl residue was performed using isatoic anhydride, the *N*-carboxyanhydride of 2-aminobenzoic acid. This compound is an activated and readily available commercial form for conducting amide couplings and esterification. The latter was done in the presence of alcohol with 4-dimethylaminopyridine (DMAP) as a catalyst in refluxing acetonitrile for one hour, as illustrated in Scheme 1.

**Scheme 2:**
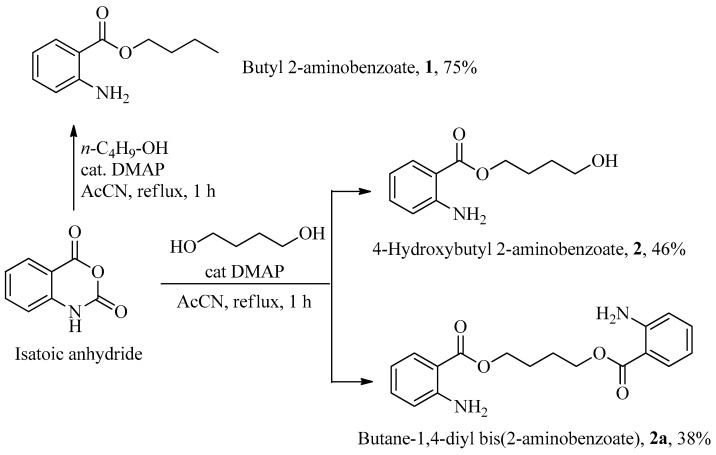
Reaction of isatoic anhydride with n-butanol and 1,4-butanediol.

Using *n*-butanol, butyl 2-aminobenzoate **1** was obtained with a 75% yield after chromatographic purification (Scheme 2). When 1,4-butanediol was used instead, a mixture of 4-hydroxybutyl 2-aminobenzoate **2** (46%) and dimeric ester butane-*bis*-2-aminobenzoate **2a**, was produced, as shown in Scheme 2. Both products were easily purified by column chromatography.

**Scheme 3:**
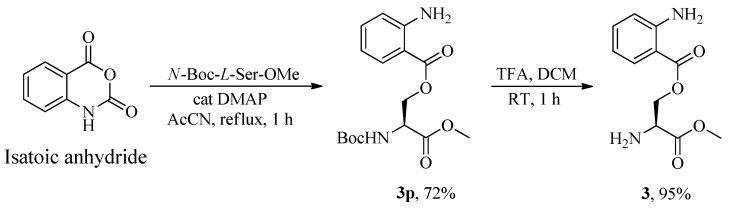
Preparation of *O*-2-aminobenzoyl-*L*-serine methyl ester.

Serine was anchored to 2-aminobenzoate starting from its *N*,*C*-protected (**p**) form, the commercially available *N*-Boc-*L*-Ser-OMe, thereby retaining the free primary alcohol function. The esterification process yielded (*S*)-2-((tert-butoxycarbonyl)amino)-3-methoxy-3-oxopropyl 2-aminobenzoate **3p** in 72% yield, which, following deprotection of the Boc group with trifluoroacetic acid in dichloromethane, produced *O*-2-aminobenzoyl-*L*-serine methyl ester **3** in 95% yield, as illustrated in Scheme 3.

**Scheme 4:**
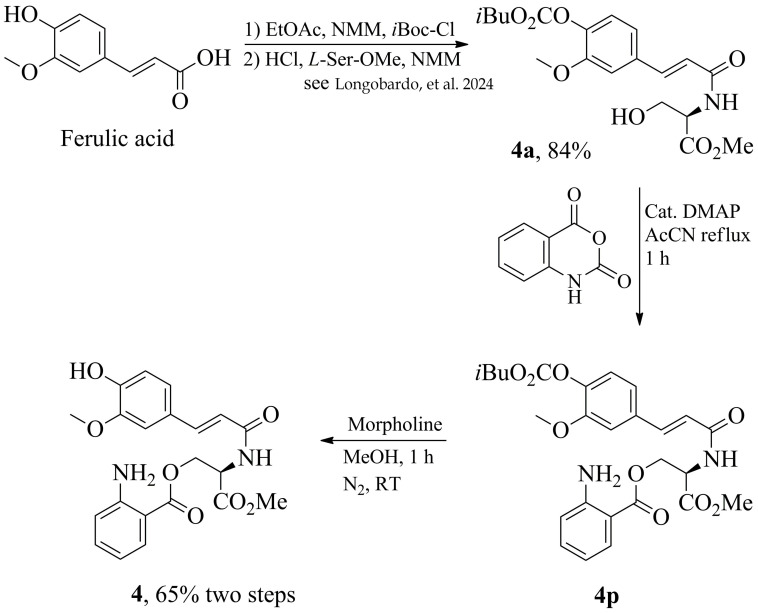
2-Aminobenzoylation of the free hydroxy group in *i*Boc-ferulic-*L*-serine methyl ester [10].

Our group recently reported [10] on an innovative and eco-friendly derivatization process for simple and chemoselective conjugation of hydroxycinnamic acids with α-amino acids. Consequently, we initially conjugated serine methyl ester with ferulic acid, producing an amide that retains a free hydroxyl group, followed by esterification with 2-aminobenzoic acid, as illustrated in Scheme 4.

Since the ferulic acid conjugated to the serine methyl ester is formed with a carbonate group protecting the phenolic function as **4a** in 84% yield, the esterification process can be conducted efficiently only on the free hydroxyl group, producing **4p**. Finally, removing the carbonate protection with morpholine in methanol at room temperature for 1 h yields, after chromatographic purification, product **4** in 65% overall yield with the free amine and phenolic function. The ^1^H- and ^13^C-NMR data of compounds **1**–**4** are reported in Tables S1–S4 of the Supplementary Material.

### 2.2. Antifungal Activity

**Figure 2:**
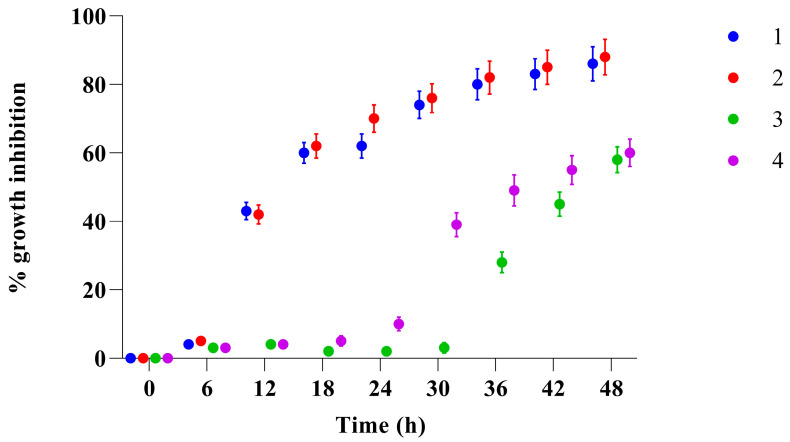
Screening of potential bioactive compounds against *Candida* growth at a concentration of 100 µg/mL.

To identify if compounds **1**–**4** had antifungal activity against *C. albicans*, first, we evaluated their inhibitory activity using only one concentration against yeast cell growth over 48 h. As shown in Figure 2, although all four compounds inhibited *Candida* growth by 50%, only the two compounds (**1** and **2**) demonstrated the ability to inhibit growth of the yeast cells by more than 80% after 48 h at the tested concentration.

**Figure 3:**
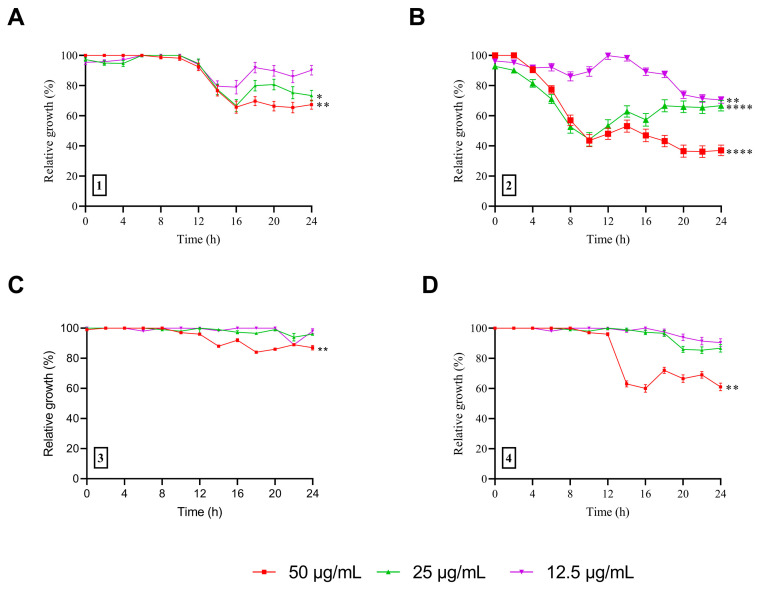
Time-kill kinetics of compounds **1**–**4** (panels (**A**–**D)**, respectively) at three different concentrations (12.5, 25, and 50 µg/mL). Asterisks indicate significant differences between treated groups compared to the control (100%), (* *p* < 0.05, ** *p* < 0.01, **** *p* < 0.0001, Tukey’s test).

In vitro time-kill studies were used to further characterize the antifungal efficacy of compounds **1**–**4** towards planktonic *Candida* cell growth, focusing on the evolving impact at different concentrations. All compounds displayed substantial concentration-dependent killing against *C. albicans*, with differential inhibitory effects on growth depending on time. As reported in Figure 3A, compound **1**, after 24 h of exposure at the highest tested concentration only reduced the growth of the yeast cells by 30% compared to the control (*p* < 0.001).

Even if only an inhibition of 30% was achieved after 24 h, the growth of *C. albicans* at the highest tested concentration was markedly retarded compared to the control. A more significant effect was observed for compound **2** (Figure 3B) after 24 h of treatment, where, even at low concentrations, it was able to inhibit the growth of *Candida* by about 25%, reaching 60% inhibition at a concentration of 50 µg/mL compared to the control (*p* < 0.0001). Compound **3** (panel C) at the highest concentration (50 µg/mL) reduces the growth of *Candida* by about 10%. Compound **4** (panel D) at the highest concentration (50 µg/mL) reduces growth by about 40%. These results, taken together, show fungistatic activity within 24 h at concentrations up to 50 µg/mL. The minimum inhibitory concentrations of compounds **1**–**4** for yeast growth were calculated using the microdilution method. Table 1 shows the obtained MIC_90_ values. The lowest MIC value of 70 µg/mL was obtained for compounds **1** and **2**, whereas the MIC values of compounds **3** and **4** were 200 and 175 µg/mL, respectively. To determine fungicidal potential, we determined the value of the minimum fungicidal concentration (MFC). From the MIC and MFC data reported in Table 1, we could conclude that only compounds **1** and **2** showed fungicidal action.

The MIC of FLC was determined according to the specific standard for in vitro susceptibility tests for *C. albicans*, confirming the Fluconazole resistance of this clinical isolate.

**Figure 4:**
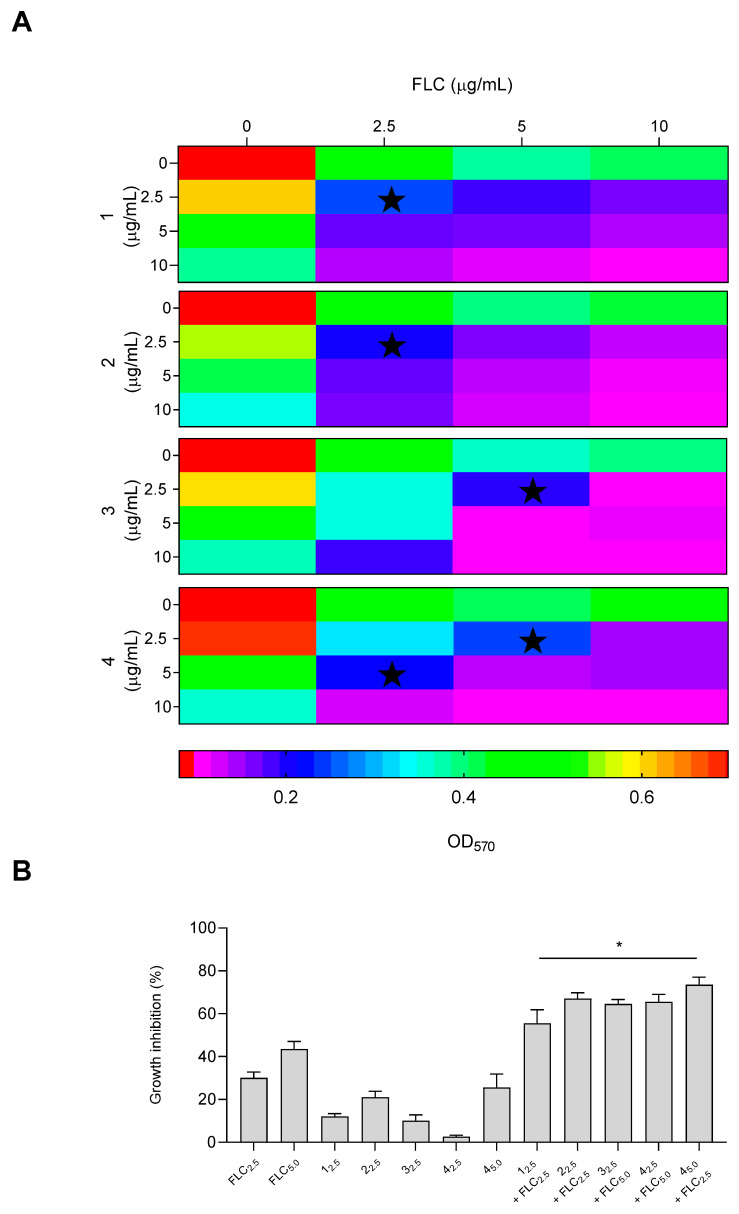
Determination of the FIC index. (**A**) Interaction of compounds **1**–**4** with antifungal fluconazole against the planktonic cells of a *C. albicans* clinical isolate. Heat map representing OD_570_ values for the interaction between compounds **1**–**4** and FLC. Stars indicate combinations with FICI ≤ 0.5, interpreted as synergistic. Exact FICI values are reported in Table S6. (**B**) Percentage of growth inhibition of *C. albicans* clinical isolate treated with compounds **1**–**4** and FLC, alone or in combination at sinergyc concentrations. Asterisks indicate significant differences between groups (* *p* < 0.05, Tukey’s test).

The interaction between compounds **1**–**4** and the antifungal drug FLC was investigated, as shown in Figure 4. The checkerboard microdilution results for all antifungal agents in combination with FLC against *C. albicans* demonstrated maximum synergy, even at low concentrations of 2.5 µg/mL for compounds **1** and **2**, with a FICI value of 0.07 (Table S6). Combination therapy offers several benefits that can address the limitations of monotherapy in treating resistant candidiasis. These advantages include increased potency, reduced drug dosages, minimized toxicity, and the ability to overcome antimicrobial drug resistance. As shown in panel B, when the two derivatives and fluconazole were used alone, they inhibited the growth of *C. albicans* by approximately 20% and 30%, respectively. Meanwhile, the combination of the derivatives/fluconazole resulted in an inhibition of approximately 60%. Compounds **3** and **4** showed synergy at concentrations of 2.5 and 5 µg/mL of fluconazole, respectively. Compound **4** also demonstrated synergy at concentrations of 5 and 2.5 µg/mL plus fluconazole, respectively, indicating the potential of using two different concentrations. The inhibition, on the other hand, reached 60% at the FICI concentrations.

Overall, the simplest ester derivatives, compounds **1** and **2**, exhibited the highest antifungal activity. In contrast, compound **3**, which bears a free amino group on the serine residue, showed reduced activity, likely due to the unprotected amino function impairing its ability to inhibit *Candida* growth. Consistently, compound **4**, in which the amino group is involved in an amide bond with the ferulic acid moiety, displayed antifungal activity comparable to that of compounds **1** and **2**, particularly at the two highest tested concentrations.

### 2.3. Biofilm Inhibition and Displacement by Compounds ***1***–***4***

It is well known that the biofilm of *C. albicans* is a distinct form of growth compared to its planktonic growth state, which plays a key role in its virulence or pathogenicity. The antibiofilm potential of compounds **1**–**4** was identified in the present investigation [4].

Hence, sub-MIC concentrations of 12.5, 25, and 50 µg/mL were selected for further evaluation on their inhibitory effects on biofilm formation of the fluconazole-resistant *C. albicans* isolate using 96-well polystyrene plates considering three different parameters. In our previous studies, this strain exhibited strong biofilm-forming ability [11].

**Figure 5:**
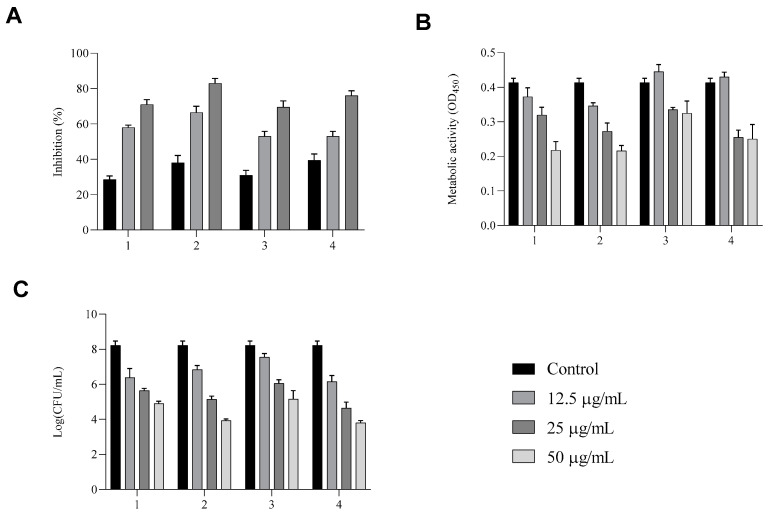
Biofilm inhibition activity. (**A**) Reduction in total biomass detected by crystal violet assay for compounds **1**–**4** (concentrations of 12.5, 25, and 50 µg/mL); (**B**) effect of compounds **1**–**4** (concentrations of 12.5, 25, 50 µg/mL) on the metabolic activity of *C. albicans* detected via XTT reduction assay; (**C**) log CFUs of *C. albicans* determined after different treatments with compounds **1**–**4** (concentrations of 12.5, 25, and 50 µg/mL) using YPD agar plates.

Here, we showed that all compounds exhibited optimal fungal biofilm inhibition (Figure 5). In panel A, the total biomass of biofilm treated with compounds **1**–**4** detected by crystal violet assay shows inhibition of about 60%, 70%, 50%, and 50%, respectively, at a concentration of 25 µg/mL and 75%, 85%, 75%, and 80%, respectively, for the highest tested concentration of 50 µg/mL.

XTT reduction assays reported in panel B show that the metabolic activity of biofilm cells was markedly decreased from 50 µg/mL for all compounds.

To complete the analysis, in addition, the anti-biofilm activity was evaluated via a viable count assay (panel C). When untreated, *C. albicans* formed dense biofilms, but in the presence of each compound at the highest concentration, we observed a log reduction of more than 2 of vital yeast cells in biofilm. These results are in line with biofilm inhibitions showing that biofilm formation was inhibited without affecting cell viability.

These results suggest strong antibiofilm activity, corroborating the idea that these compounds, are less likely than conventional antifungal agents to promote the development of drug resistance, as they do not target planktonic cell growth. Furthermore, their antibiofilm effects appear to be driven by mechanisms distinct from their antifungal properties.

**Figure 6:**
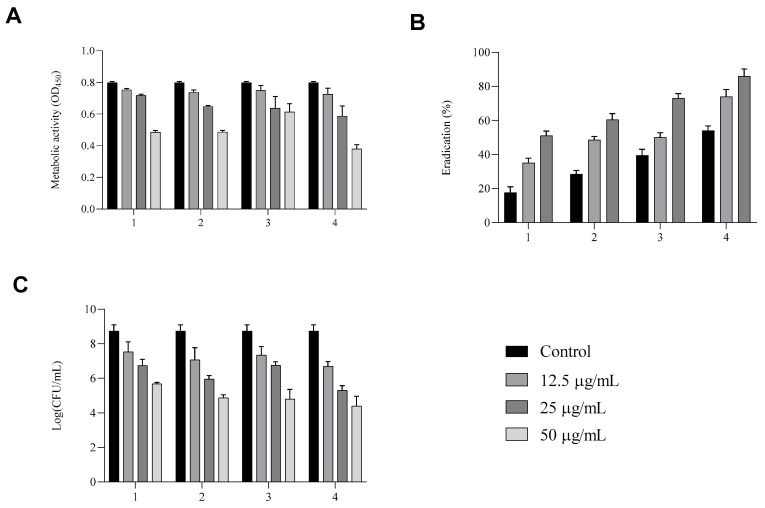
Biofilm eradication activity; (**A**): Reduction in total biomass detected by crystal violet assay for compounds **1**–**4** (concentrations of 12.5, 25, and 50 µg/mL); (**B**): effect compounds **1**–**4** (concentrations of 12.5, 25, 50 µg/mL) on the metabolic activity of *C. albicans* detected via XTT reduction assay; (**C**): log CFUs of *C. albicans* determined after different treatments with compounds **1**–**4** (concentrations of 12.5, 25, and 50 µg/mL) using YPD agar plates.

We speculate that compounds **1**–**4** had a destructive effect on the 24 h pre-formed biofilms of *C. albicans* (Figure 6A–C). As shown in panel A, biofilms produced and posteriorly treated with compounds **1**–**4** for an additional 24 h presented a reduction in the total biomass detected by CV assay only at a concentration of 50 µg/mL. Eradication ability was also investigated by studying the metabolic activity of *Candida* cells in biofilm after treatment. As shown in panel B, compounds **1** and **2** showed a clear dose-dependent reduction in metabolic activity, with the lowest OD_450_ values observed at the highest concentration, indicating strong antifungal activity. In contrast, compound **3** was less effective, showing a moderate reduction in metabolic activity compared to the untreated control. Interestingly, compound **4** produced a reduction in metabolic activity comparable to that of compounds **1** and **2** at higher concentrations, supporting its potential as an effective antifungal agent.

The viability of yeasts cells after treatments was also determined. Compounds **1**, **2**, and **4** induced a marked decrease in CFU/mL, whereas compound **3** was less effective.

Compound **4** penetrated efficiently into the interior of the biofilm, and its penetration ability was enhanced increased concentration.

### 2.4. Expression Analysis of the Effects of Compounds ***1***–***4*** on ERG11, HWP1, SAP1, SAP2, SAP6, and ALS3 of C. albicans Biofilm Formation by RT-PCR

**Figure 7:**
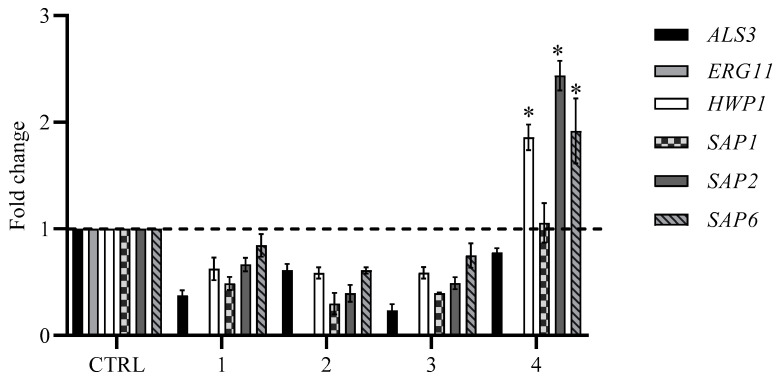
Differential gene expressions of the effects of compounds **1**–**4** on *C. albicans*. Fold changes represent the transcriptional variances identified in cells subjected to treatment compared to untreated cells, as determined by qRT-PCR. *C. albicans* cells were treated with concentrations of 12.5 µg/mL for 18 h. Dashed line represents the baseline expression level (fold change = 1) in the control condition. Act1 was used as the housekeeping gene. *, *p* < 0.05 vs. untreated controls (Holm-Sidak test).

We further investigated whether the observed inhibitory effect on biofilm formation was mediated by changes in gene expression. Since the yeast-to-hyphae transition and biofilm formation are important events in the virulence of fungus, genes involved in this transition were primarily studied. RNA was extracted from *C. albicans* biofilm treated with 12.5 µg/mL of each compound, and the gene expression of *ERG11*, *HWP1*, *SAP1*, *SAP2*, *SAP6*, and *ALS3* was examined. Our findings confirm that the expression levels underwent to changes and, indeed, vary between different compounds. The initial step in biofilm formation is adhesion, which is mediated by *als* genes and the *hwp1* gene, which plays an important role in hyphal growth and biofilm formation. The treatment with compounds **1**–**3** completely inhibited the expression of the *ALS3* and *HWP1* genes (Figure 7), while in presence of compound **4**, we observed an upregulation of *HWP1* and an insignificant downregulation of *ALS3*. The expression of the *SAP* genes that play a crucial role in the pathogenesis and are the most common genes expressed during *Candida* infection were downregulated in cells treated with the first three compounds; only compound **4** showed a significant upregulation of *SAP2* and *SAP6*. *ERG11* was not detected any samples.

### 2.5. In Vitro Cytotoxicity Investigation of Compounds ***1***–***4*** of HaCaT Cell Line

**Figure 8:**
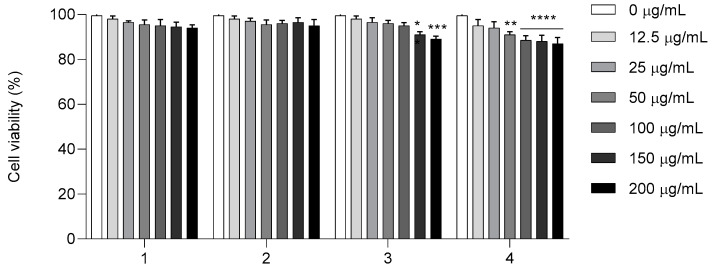
In vitro cytotoxicity investigation of compounds **1**–**4** in the HaCaT cell line (* *p* < 0.05, ** *p* < 0.01, *** *p* < 0.001 **** *p* < 0.0001, Tukey’s test).

Finally, to confirm the potential application of compounds **1**–**4**, cytotoxic studies of the HaCaT cell line were performed (Figure 8). Cell viability was assessed in the HaCaT cell line after 24 h of exposure to compounds **1**–**4** in a concentration range of 12.5 to 200 μg/mL. As shown in Figure 8, the MTT assay revealed that, up to a concentration of 200 μg/mL, compounds **1**–**2** were both non-cytotoxic. These results show that compounds **1** and **2** were not harmful to human HaCaT cells, even at a concentration higher than the MIC and MFC for the *Candida* isolate, exhibiting low toxicity. Compound **3** at concentrations of 150 and 200 μg/mL and compound **4** at concentrations of 100, 150, and 200 μg/mL inhibited 20% of cells, showing slightly decreased viability but no considerable toxic activity compared to the control.

### 2.6. In Vivo Toxicity Investigation of Compounds ***1***–***4*** in Caenorhabditis Elegans and Genotoxicity with Vicia Faba

**Figure 9:**
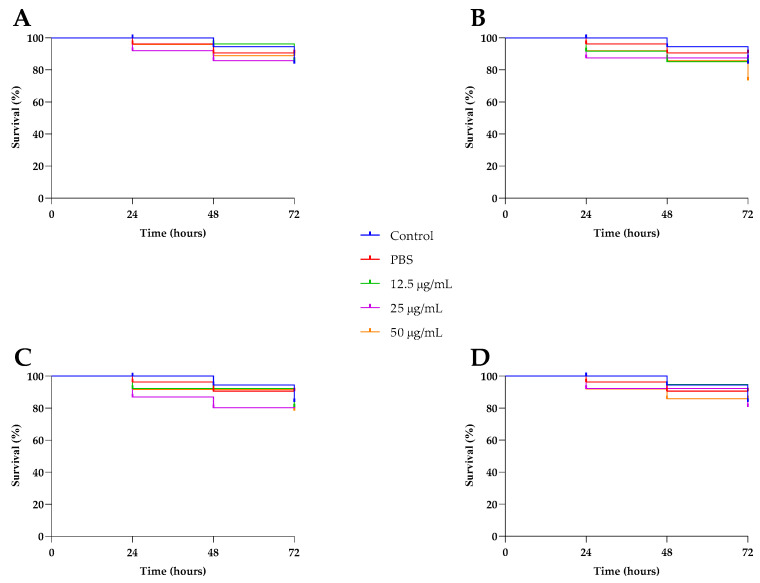
Survival curves of *C. elegans* after 72 h of exposure to tested compound concentrations ranging from 12.5 to 50 μg/mL. (**A**–**D**) Compounds **1**–**4**, respectively.

**Figure 10:**
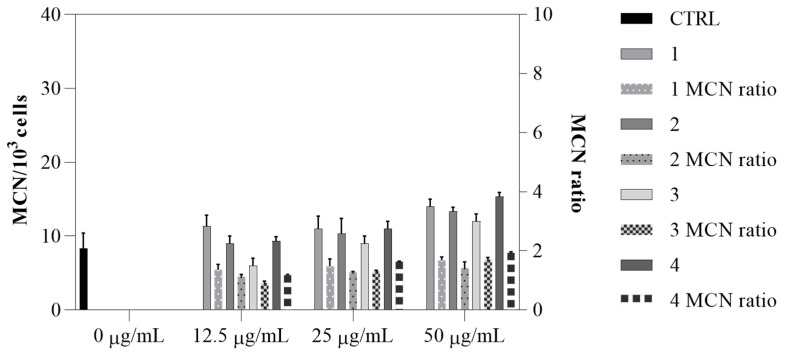
Micronucleus assay in *V. faba* after 72 h of exposure to compounds **1**–**4** at 12.5, 25, and 50 μg/mL. The number of micronuclei (*MCN*, expressed as 10^3^ cells) is represented in light gray, while the Micronucleated Cell Ratio (MCN Ratio) is shown in black.

Invertebrate hosts have been successfully used to study the toxicity and effectiveness of various substances. Therefore, we assessed the toxicity of compounds **1**–**4** in *Caenorhabditis elegans* at sub-MIC concentrations of 12.5, 25, and 50 μg/mL. As shown in Figure 9, no significant differences were observed between the untreated larvae and the treated worms, confirming that the compounds were not toxic to *C. elegans* at the tested concentrations. The safety of compounds **1**–**4** was also evaluated using a *Vicia faba* micronucleus assay. The analysis revealed no significant increase in micronucleus frequency at the tested concentrations (12.5, 25, and 50 μg/mL) (Figure 10), with values ranging from 8 to 14. Additionally, the genotoxic ratio did not exceed the threshold value of 2, indicating the absence of genotoxic effects. All these data indicate the efficacy and possible safe use of all four compounds.

## 3. Discussion

*Candida* species can shift from harmless commensals to pathogenic organisms under conditions such as excessive antibiotic use, immune suppression, or dysfunction of mucosal barriers. Unfortunately, existing antifungal treatments are limited in their effectiveness and come with side effects, which has led to growing interest in alternative therapies, especially those targeting fungal biofilms [12,13]. *C. albicans* exhibiting biofilm morphology, that is one of the major virulence factors, displays a high degree of resistance to a wide range of antifungal drugs, posing a significant challenge to clinical treatment. As a result, the development and design of antifungal agents must not only show strong efficacy against fungi but also have the capacity to prevent biofilm formation and aid in its eradication.

2-Aminobenzoic acid and its derivatives, which are found in many plants, are frequently assessed for their pharmacological activity and used as model compounds in the development of promising bioactive substances. Among these synthetic compounds, esters and amides are particularly noteworthy due to their broad range of biological activities. In fact, anthranilic acid and its derivatives demonstrate a wide array of biological effects, including anti-inflammatory, antioxidant, anticancer, hypoglycemic, cytoprotective, antidepressant, antibacterial, antifungal, antimalarial, cytotoxic antitumor, and phytotoxic properties [14].

This study highlights the promising potential of four 2-aminobenzoic acid derivatives (compounds **1**–**4**) in combating biofilm formation and antimicrobial resistance in an ocular clinical isolate of *C. albicans*, a pathogen known for its strong ability to form biofilms and its resistance to standard antifungal drugs. These infections are usually treated with azole antifungals, but the emergence of drug-resistant strains has reduced their effectiveness.

Fluconazole-resistant *C. albicans* has emerged as a significant ocular healthcare-associated pathogen. This study introduces molecules that enhance the action of fluconazole, restoring its effectiveness against azole-resistant *Candida* strains.

Here, analysis of the in vitro antifungal activity of compounds **1**–**4** and their combination with FLZ showed efficacy against the clinical isolate. Compounds **1** and **2** showed the lowest MIC of 70 µg/mL, while compounds **3** and **4** required higher concentrations. Only compounds **1** and **2** exhibited fungicidal activity. These results confirm that in the case of antifungal activity against *C. albicans*, anthranilic acid requires esterification, as reported previously [15].

The presence of the OH group in compound **2** enhanced its pharmacological activity, despite a decrease in lipophilicity, which results in reduced penetration of the compound into biological membranes. Numerous studies [16] have extensively explored lipophilicity, and it is considered one of the most important factors influencing antifungal activity. The introduction of an aromatic ring in compound **4** led to a decrease in antifungal activity. The molecular volume of these groups may have contributed to this change in biological potency.

The combination of these compounds with fluconazole showed strong synergy, reducing fungal load and overcoming drug resistance. The combination therapy significantly increased potency, reduced dosages, and achieved up to 60% inhibition of *C. albicans*. Differences in the FLC potentiation activity of compounds **1**–**4** may be attributed to differences in interaction with structural components of the cell wall.

This synergistic effect is particularly relevant, given the increasing prevalence of fluconazole-resistant strains and the limited therapeutic options available. The compounds may act on different fungal targets or cellular pathways than fluconazole, such as membrane integrity, oxidative stress responses, or biofilm matrix disruption. These complementary mechanisms could explain the enhanced efficacy observed in combination treatments. Understanding and exploiting such synergistic interactions is crucial for the development of new therapeutic strategies to restore antifungal susceptibility and improve clinical outcomes [17].

The antibiofilm potential of compounds **1**–**4** against a fluconazole-resistant *C. albicans* strain was evaluated using sub-MIC concentrations of 12.5, 25, and 50 µg/mL. All compounds effectively inhibited biofilm formation, with the highest concentrations (50 µg/mL) showing 75–85% inhibition of biofilm biomass. XTT assays revealed a significant decrease in metabolic activity, and viable count assays showed over 2 log reductions in viable yeast cells in biofilms. These results suggest that compounds **1**–**4** exhibit strong antibiofilm activity without affecting cell viability, potentially reducing drug resistance compared to conventional antifungals. Compound **4** demonstrated the highest eradication ability, reaching 80% inhibition and showing good penetration into the biofilm.

Various natural and synthetic compounds have been shown to inhibit *Candida albicans* biofilm formation by downregulating multiple hypha-specific and biofilm-associated genes [18].

Among the numerous virulence determinants of *Candida* species, the *HWP1***,** *ALS3*, and *SAP1* genes are critically involved in the initial stages of biofilm development and ERG11 in the expression of resistance to azoles. The *HWP1* gene encodes a mannose-rich cell wall protein that is indispensable for hyphal morphogenesis. The yeast-to-hyphae transition is a key virulence trait associated with enhanced pathogenicity in systemic infections such as candidemia. The *ALS3* gene encodes an adhesin protein that mediates fungal adherence to host cells and is implicated in clathrin-mediated endocytosis via interaction with host cell adhesion molecules E-cadherin and N-cadherin. In vitro evidence has established a positive correlation between *ALS3* expression and epithelial cell damage, and the gene also contributes to interspecies co-adhesion during infections involving *C. albicans*. Members of the *SAP* gene family have been reported to exhibit differential expression under various in vitro environmental conditions [19,20], leading to the hypothesis that distinct *SAP* genes may also be differentially regulated in vivo, thereby contributing to the pathogenesis of diverse *Candida albicans* infections. Consequently, secreted aspartyl proteinases or their corresponding genes (the *SAP* gene family) are actively expressed during the infectious process. *SAP1* facilitates the degradation of host cell membranes and extracellular matrix components, compromising tissue integrity and promoting fungal invasion of host tissues, while *SAP2–6* genes are also regulated during hyphal development.

The RT-PCR analysis concludes that the four compounds target virulence genes that are involved in the hyphal and biofilm formation (virulence factors) in the clinical isolate. Our results show that these compounds have varying effects on key genes involved in biofilm formation in *C. albicans*. The downregulation of *HWP1* by compounds **1**–**3** indicates that these compounds interfere with critical pathways related to hyphal development and growth, which are essential for biofilm formation and the pathogenicity of *C. albicans*. This suppression may hinder the formation of strong biofilms, reducing the fungus’s ability to cause infection. On the other hand, compound **4** had no effect on the expression of these genes, suggesting it may inhibit biofilm formation through different mechanisms, such as by disrupting cell membrane integrity or affecting extracellular matrix components. All the tested compounds were found to downregulate *ALS3*, pointing to the involvement of this gene in the inhibitory effect. Additionally, the expression profile of *SAP*, one of the most expressed genes during infection, was also altered in different ways; it was downregulated by compounds **1**–**3**, while compound **4** significantly upregulated *SAP2* and *SAP6*.

Cytotoxicity studies of HaCaT cells showed that compounds **1** and **2** were non-cytotoxic up to 200 μg/mL, even at concentrations higher than their MIC and MFC for *Candida*. Compounds **3** and **4** caused a slight reduction in cell viability at higher concentrations (150–200 μg/mL) but showed no significant toxic effects compared to the control.

These 2-aminobenzoic derivative molecules were non-toxic to *C. elegans* and showed an absence of genotoxic effects in a *V. faba* micronucleus assay.

In conclusion, our data suggest that these compounds might be useful in the development of novel antifungal formulations with improved antimicrobial properties to treat biofilm infections caused by *C. albicans*.

## 4. Materials and Methods

### 4.1. Strain and Culture Conditions

A clinical ocular *C. albicans* strain was previously isolated from infected patients and maintained on Yeast Peptone Dextrose Agar (YPD, Sigma-Aldrich, Wien, Austria) in our laboratory collection. The susceptibility to fluconazole of *C. albicans* clinical isolated was determined using the disc diffusion method as described by the Clinical and Laboratory Standards Institute [21] and reported in our previous study [11]. The strain was cultured in Roswell Park Memorial Institute medium (RPMI 1640), supplemented with *L*-glutamine, and adjusted to pH 7.4 using 0.165 mol/L of morpholino-propanesulfonic acid (MOPS) (Sigma Chemical Co., St. Louis, MO, USA), following the Clinical and Laboratory Standards Institute (CLSI) M27-A3 guidelines [22].

### 4.2. Synthesis, Purification, and Characterization of Compounds

Unless otherwise specified, all reactions were performed using dried solvents. Chemicals and solvents were purchased from TCI Eu and used as received. Identities of products were confirmed using ^1^H, ^13^C spectra, and HR-MS. The reactions were followed by TLC using silica gel-precoated aluminum sheets (Macherey Nagel, Düren, Germany: Alugram Xtra SIL GEL UV254 Nr. 818333, thickness 0.2 mm). TLC plates were analyzed under a UV lamp (254 nm). Column chromatography was performed using 63–200 mm high-purity silica gel and EtOAc-Hexane as eluent. NMR spectra were recorded in CDCl_3,_ DMSO-*d*_6_, or CD_3_OD on a Bruker Avance 400 (400 MHz for ^1^H, 101 MHz for ^13^C) (Bruker, Billerica, MA, USA). MALDI-TOF mass spectrometric analyses were conducted on a Voyager-De Pro MALDI mass spectrometer (PerSeptive Biosystems, Framingham, MA, USA).

### 4.3. Compound Screening Assay and Time-Kill Assay

To rapidly assess the inhibitory effects on fungal growth or viability, *C. albicans* cells (10^5^ cells/mL) were grown in triplicate in a 96-well plate in RPMI medium at 30 °C for 48 h, using a single concentration of 100 µg/mL for each compound. The optical density at 600 nm (OD_600_) was measured every 10 min using a microtiter plate reader (Varioskan LUX Multimode Microplate Reader ThermoFisher Scientific, Waltham, MA, USA). The percentage growth of each compound was calculated relative to the untreated wells. All compounds displayed more than 50% growth inhibition, so they were selected and underwent a time-kill assay to confirm bioactivity. Concentrations of fungal cells corresponding to an inoculum of 1 × 10^6^ CFU/mL were added to each 96-well plate, in addition to different concentrations of each bioactive molecule: 12.5, 25, and 50 µg/mL. Plates were incubated in the plate reader at 30 °C, and OD_600_ readings were taken every 10 min over 24 h. Each experiment was performed in triplicate. For each concentration, time-kill curves were produced, and relative growth was calculated as the percentage of OD_600_ measurements of fungally treated cells relative to that of the untreated control.

### 4.4. Determination of Minimal Inhibitory Concentration and Minimal Fungicidal Concentration

The Clinical and Laboratory Standards Institute broth dilution method was used to determine minimum inhibitory concentrations (MICs) of planktonic cell growth [22]. Briefly, an overnight cultivation of *C. albicans* was diluted at a concentration of 1 × 10 ^6^ cells/mL in RPMI-1640 (ThermoFisher Scientific Inc., Waltham, MA, USA) and dispensed into wells of a 96-well polystyrene plate containing different concentrations of the compounds ranging from 5 to 200 µg/mL, then incubated for 24 h at 37 °C. MIC was defined as the lowest concentration at which planktonic cell growth was undetectable. To assess the fungicidal effect of the compounds on *C. albicans*, additionally, 10 μL was removed from each well without visible growth after the MIC assay, cultured on YPD plates, and incubated for 24 h at 37 °C. Colony forming units (CFU) on the plates were counted after incubation, and CFU/mL was calculated using the following formula: CFU/mL = Number of colony units × dilution factor/volume plated (mL). The minimum fungicidal concentration (MFC) was determined as the lowest concentration of the two compounds capable of preventing 100% fungal growth after plating. The compound was considered fungicidal if the MFC/MIC ratio was ≤4 and fungistatic if the ratio was ≥4 [23].

### 4.5. FICI Index Determination for Compounds **1**–**4** and Fluconazole

The effects of the combination of the four compounds with conventional antifungal fluconazole on *C. albicans* cells were determined by checkerboard microtiter assay, as described previously [24].

To compare compounds **1**–**4**, the drugs were applied horizontally and vertically in serial dilutions to generate combinations of antifungal agents at different ratios. Each test plate also contained drug-free control wells with medium only. A volume of 100 μL of prepared yeast inoculum (10^5^ CFU/mL) was applied vertically to the wells of this same plate. Results were then analyzed after a 24 h incubation at 35 ± 2 °C. The FICI was calculated for each agent by dividing the inhibition concentration of the antifungal combination by its MIC value. The calculation formula for the FICI model is expressed as follows: FICI = *Ac*/*Aa* + *Bc*/*Ba*, where *Ac* and *Bc* are the MIC values of the tested agents in combination, while *Aa* and *Ba* correspond to these values for single-agent A and B treatments. A FICI ≤0.5 indicates synergy, 0.5 < FICI ≤ 4 corresponds to no interaction, and FICI > 4 indicates antagonism [25]. Experiments were performed in triplicate.

### 4.6. Biofilm Inhibition and Eradication Assays

*C. albicans* biofilms were developed on 96-well plates, as previously reported [11]. Briefly, to evaluate the MBIC ability of the four compounds, their different sub-MIC concentrations, ranging from 12.5 to 50 μg/mL, were added to 10^6^ CFU/mL *Candida* cells in 96-well plates for 24 h at 37 °C. To detect total biomass, biofilm cells on the well surfaces were subsequently stained with crystal violet (0.1% *w*/*v*, Sigma-Aldrich, St. Louis, MO, USA) for 20 min and washed three times with distilled water. The crystal violet was dissolved using 95% ethanol, and the absorbances of crystal violet in ethanol were measured at 570 nm using a spectrophotometer [26]. To detect cell viability in biofilms, the formed biofilms were scraped off from each well and placed in a microtube filled with medium to obtain a biofilm suspension that was subsequently diluted and, finally, inoculated on YPD agar plates and incubated at 37 °C for 24 h. The colony forming units per well (CFU/well) were counted as reported above. The metabolic activities of yeast cells within biofilms were measured using a tetrazolium 2,3-*bis*(2-methoxy-4-nitro-5-sulfophenyl)-5-[(phenylamine)carbonyl]-2H-hydroxide reduction assay (XTT) (Sigma-Aldrich, St. Louis, MO, USA) according to the manufacturer’s method, and the colorimetric change was measured at 450 nm using a microtiter plate reader (Varioskan LUX Multimode Microplate Reader Thermofisher Scientific USA). For the biofilm eradication analysis, established biofilms of *C. albicans* were formed for 24 h. Compounds **1**–**4** were then introduced at the same sub-MIC concentrations (12.5, 25, and 50 μg/mL), and after an additional 24 h of incubation, their biofilm eradication ability was measured as described above. Two independent experiments with six replicates were performed at each concentration for both inhibition and eradication assays and for each detection method.

### 4.7. RNA Isolation and Quantitative Real-Time PCR (qRT-PCR)

Total RNA was extracted from *C. albicans* culture grown for 18 h at 37 °C in the absence and presence of compounds **1**–**4** (12.5 µg/mL) using a Direct-zol^TM^ RNA Miniprep Plus Kit (ZYMO RESEARCH, Irvine, CA, USA) according to the manufacturer’s protocol. Subsequently, RNA samples were reverse-transcribed into cDNA using an iScript cDNA Synthesis kit (Bio-Rad, Milan, Italy). Quantitative PCR was then carried out with a Sensifast SYBR Lo-ROX kit (Meridian Bioscience, Cincinnati, OH, USA) using a thermal cycler (Agilent Technologies, Inc., Milan, Italy). In brief, 25 µL of SYBR Green was combined with 100 ng of cDNA, 1 µM of each primer, and 12.5 µL of QuantiFast SYBR Green PCR Master Mix (2×). The thermal cycler used the following thermal profile: an initial denaturation step at 95 °C for 10 min, followed by 40 amplification cycles consisting of 15 s at 95 °C and 1 min at 60 °C, a final elongation step at 95 °C for 15 s, and one cycle for melting curve analysis (ranging from 60 °C to 95 °C) to confirm the presence of a single product. Each reaction included a no-template control for each primer pair reported in Table S5. To assess intra-assay variability, all reactions were performed in triplicate. Fluorescence was measured using StepOnePlus Real Time PCR systems software v2.3 (ThermoFisher Scientific, Waltham, MA, USA). Gene expression levels were analyzed using the 2^−ΔΔCT^ method, where the CT value represents the average threshold cycle from three independent experiments. Results are expressed as the fold change in gene expression, normalized to the *Act1* gene, which was used as an internal control [27,28].

### 4.8. Cell Culture Conditions and Cell Cytotoxicity Assay

HaCAT cells (non-tumorigenic human keratinocyte cells) were grown in Dulbecco’s Modified Eagle Medium (DMEM, Sigma Aldrich, St. Louis, MO, USA), supplemented with 10% fetal bovine serum, 1% l-glutamine, and 1% penicillin/streptomycin (Sigma Aldrich) in a humidified incubator at 37 °C and 5% CO_2_. The cytotoxicities of compounds **1**–**4** were assessed using 3-(4,5-dimethylthiazol-2-yl)-2,5-diphenyltetrazolium bromide (MTT assay). The concentrations of the four compounds used in this study were tested. First, 2.5 × 10^5^ cells/mL (100 μL) were seeded in 96-well culture plates, treated, and incubated for 24 h. After incubation, 100 µL of MTT solution (5 mg/mL) (Sigma-Aldrich, St. Louis, MO, USA) was added to each well, and the plate was incubated at 37 °C/5% CO_2_ for 4 h. To dissolve the formazan crystals, 100 µL of DMSO (Sigma-Aldrich) was added, and the absorbance was detected at 570 nm in a microplate reader. The cell viability (%) was calculated as follows: (OD of treated cells/OD of control) × 100. As an internal control, cells containing only DMSO as the solvent at the same concentration used in each test wells were selected. The tests were performed in triplicate.

### 4.9. Toxicity Assays Using the Nematode Model and a Plant Germination Model

To investigate the toxicities of compounds **1**–**4**, the *C*. *elegans* strain was used, as previously described [29]. Briefly, synchronized young adult nematodes were cleaned twice with M9 buffer (3 g/L KH_2_PO_4_, 6 g/L Na_2_HPO_4_, 5 g/L NaCl, and 1 mM MgSO_4_), and ~30 worms were dispensed into each well of 96-well plates containing M9 buffer (200 μL) and compounds **1**–**4** (12.5, 25, 50 µg/mL). Plates were then incubated for 72 h at 25 °C without agitation under dark conditions. The survival of nematodes was detected by counting dead worms under a microscope (40 × magnification). Two independent experiments with six replicates were performed at each concentration. Results are shown using the Kaplan–Meier method.

### 4.10. Genotoxicity Assays Using the Seed Germination Model with V. faba

A toxicity assessment with *V. faba* was performed following ISO guidelines 29200:2013 [30]. For this purpose, macrophyte seeds (n = 5) were placed on Whatman No. 1 filter paper, which had been saturated with 6 mL of different test solutions at three concentrations (12.5, 25, and 50 µg/mL). The exposure was conducted in triplicate using *Petri* dishes to ensure reproducibility. The seeds were incubated in complete darkness at a temperature of 22 ± 2 °C for 96 h. After the incubation period, the root tips of the germinated seeds were carefully excised and fixed in a 1:3 acetic acid:ethanol solution for 24 h. Subsequently, the root tips were stained using Schiff’s reagent following the Feulgen method, then gently squashed onto microscope slides for analysis. The frequency of micronuclei (MCN%) was quantified by analyzing 10^3^ root-tip cells of *V. faba*, with image analysis performed using ImageJ software version 1.53t. The micronucleated cell ratio (MCN ratio) was calculated as the ratio of the number of cells with micronuclei to the total number of cells analyzed. If the MCN ratio exceeds a value of 2, the sample is considered genotoxic.

### 4.11. Statistical Analysis

Statistical analysis was performed using GraphPad Prism version 8.4.2. Data are expressed as the mean ± standard deviation (SD) from at least two or three independent experiments. For comparisons among multiple groups, one-way or two-way analysis of variance (ANOVA) was conducted, followed by Tukey’s test for multiple comparisons. Molecular analysis was evaluated using the Holm–Sidak test for multiple comparisons. For the toxicity assay on *C. elegans*, survival curves were generated using the Kaplan–Meier method, and statistical significance was determined with the log-rank test.

## Figures and Tables

**Table 1:** Evaluation of the minimum concentration values inhibiting fungal growth and the minimum fungicidal concentration expressed in µg/mL for compounds **1**–**4**, as well as for fluconazole against a *C. albicans* clinical isolate. The values were obtained from a minimum of three independent experiments.

	MIC	MFC	MFC/MIC	
	(µg/mL)		Ratio	
1	70	150	2.14	fungicidal
2	70	150	2.14	fungicidal
3	200	>200	-	-
4	175	>200	-	-
FLC	64	Nt	-	-

## Data Availability

The original contributions presented in the study are included in the article. Further inquiries can be directed to the corresponding author.

## Supplementary Materials

The following supporting information can be downloaded at https://www.mdpi.com/article/10.3390/antibiotics14050432/s1: Table S1: ^1^H, ^13^C, and 2D NMR data of **1** in CDCl_3_. Table S2: ^1^H, ^13^C, and 2D NMR data of **2** in CDCl_3_. Table S3: ^1^H, ^13^C, and 2D NMR data of **3** in CDCl_3_. Table S4: ^1^H, ^13^C, and 2D NMR data of **4** in CDCl_3_. Table S5: Primer sequence. Table S6. Fractional inhibitory concentration index (FICI) values for the combinations of compounds **1**–**4** with fluconazole against *Candida albicans* clinical isolate.
