# Supplementary material for: Antifungal and Antibiofilm Activities of 2-Aminobenzoic Acid Derivatives Against a Clinical Ocular Candida albicans Isolate for Biomedical Applications
Source: Antibiotics (Basel). 2025 Apr 25;14(5):432. doi: 10.3390/antibiotics14050432 (PMC12108512; doi:10.3390/antibiotics14050432)
Supplement: Supplementary file 1 [file antibiotics-14-00432-s001.zip › antibiotics-3589098-supplementary.pdf]

Spectral data

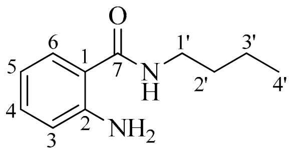

2-Amino-*N*-butylbenzamide. White powder. ^{1}H- and ^{13}C-NMR: see Table S1. MS-TOF (positive ions): $[M]^+$ calculated for $C_{11}H_{16}N_2O$: *m*/*z* 192.1263; found 193.2576 $[M + H]^+$ (64%).

Table S1. ^{1}H, ^{13}C and 2D NMR data of **1** in $CDCl_3$.

Position	Residue	$^{13}C^a$	$^1H^a$, multiplicity (J in Hz)	^1H-^1H COSY	^1H-^{13}C HMBC
1	C	111.12	-		
2	C	150.42	-		
3	CH	116.65	6.68, d (8.4)	7.28	150.42, 116.24, 111.12
4	CH	133.95	7.28, t (8.3)	6.68, 6.67	150.42, 131.20
5	CH	116.24	6.67, t (8.3)	7.89, 7.28	116.65, 111.12
6	CH	131.20	7.89, d (8.4)	6.67	168.23, 150.42, 133.95
7	C	168.23	-		
1′	CH_2	64.14	4.30, t (6.6)	1.50	168.23, 30.76, 19.29
2′	CH_2	19.29	1.50, m	4.30, 1.78	64.14, 30.76, 13.73
3′	CH_2	30.76	1.78, m	1.50, 1.00	64.14, 19.29, 13.73
4′	CH_3	13.73	1.00, t (7.4)	1.78	30.76, 19.29

[a]Chemical shifts in ppm.

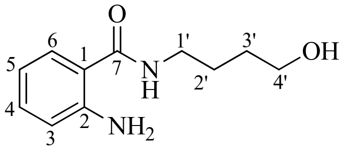

2-Amino-*N*-(4-hydroxybutyl)benzamide. White powder. ^1H- and ^{13}C-NMR: see Table S2. MS-TOF (positive ions): $[M]^+$ calculated for $C_{11}H_{16}N_2O_2$: *m/z* 208.1212; found 209.2570 $[M + H]^+$ (58%).

Table S2. ^1H, ^{13}C and 2D NMR data of **2** in $CDCl_3$.

Position	Residue	$^{13}C^a$	$^1H^a$, multiplicity (J in Hz)	^1H-^1H COSY	^1H-^{13}C HMBC
1	C	110.85	-		
2	C	150.44	-		
3	CH	116.22*	6.68, d (8.2)	7.28	150.44, 116.64, 110.85
4	CH	134.02	7.28, dd (7.6; 1.6)	6.68, 6.65	150.44, 131.09
5	CH	116.64*	6.65, dt (8.1; 1.0)	7.88, 7.28	116.22, 110.85
6	CH	131.09	7.88, dd (8.0; 1.5)	7.28, 6.65	168.11, 150.44, 134.02
7	C	168.11	-		
1′	CH_2	64.02	4.34, t (6.4)	1.89	168.11, 29.24, 25.19
2′	CH_2	25.19	1.89, m	4.34, 1.76	64.02, 62.41, 29.24
3′	CH_2	29.24	1.76, m	3.75, 1.89	64.02, 62.41, 25.19
4′	CH_2	62.41	3.75, t (6.4)	1.76	29.24, 25.19

[a]Chemical shifts in ppm. *Interchangeable values

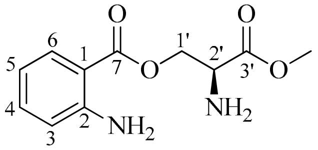

(*S*)-2-Amino-3-methoxy-3-oxopropyl 2-aminobenzoate. White powder. ^{1}H- and ^{13}C-NMR: see Table S3. MS-TOF (positive ions): $[M]^+$ calculated for $C_{11}H_{14}N_2O_4$: *m*/*z* 238.0954; found 239.2401 $[M + H]^+$ (67%).

Table S3. ^{1}H, ^{13}C and 2D NMR data of **3** in $CDCl_3$.

Position	Residue	$^{13}C^a$	$^1H^a$, multiplicity (J in Hz)	^1H-^1H COSY	^1H-^{13}C HMBC
1	C	110.10	-		
2	C	150.62	-		
3	CH	116.35*	6.66, d (8.3)	7.28	116.70, 110.10
4	CH	134.42	7.28, dd (8.0; 1.2)	6.66, 6.64	150.62, 131.12
5	CH	116.70*	6.64, t (7.9)	7.80, 7.28	116.35, 110.10
6	CH	131.12	7.80, d (8.1)	6.64	167.54, 150.62, 134.42
7	C	167.54	-		
1′	CH_2	66.15	4.51, d (4.6)	3.88	173.56, 167.54
2′	CH	53.78	3.88, t (4.6)	4.51	173.56, 66.15
3′	C	173.56	-		
OCH_3	CH_3	52.48	3.78, s		173.56

[a]Chemical shifts in ppm. *Interchangeable values

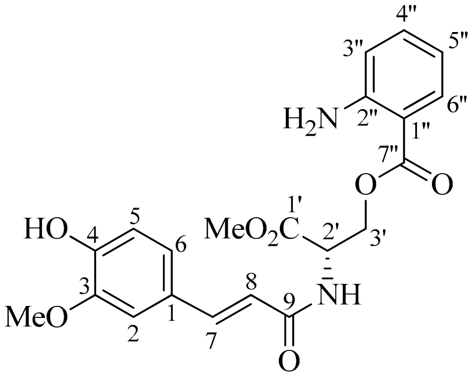

(*S*,*E*)-2-(3-(4-Hydroxy-3-methoxyphenyl)acrylamido)-3-methoxy-3-oxopropyl 2-aminobenzoate. White powder. ^{1}H- and ^{13}C-NMR: see Table S4. MS-TOF (positive ions): $[M]^+$ calculated for $C_{21}H_{22}N_2O_7$: *m/z* 414.1427; found 415.4088 $[M + H]^+$ (71%).

Table S4. ^{1}H, ^{13}C and 2D NMR data of **4** in $CDCl_3$.

Position	Residue	$^{13}C^a$	$^{1}H^a$, multiplicity (J in Hz)	^{1}H-^{1}H COSY	^{1}H-^{13}C HMBC
1	C	127.10	-		
2	CH	109.44	6.98, s		147.62, 142.30, 122.66
3	C	146.70	-		
4	C	147.62	-		
5	CH	114.69	6.87, d (8.1)	7.03	146.70, 127.10
6	CH	122.66	7.03, d (8.1)	6.87	147.62, 142.30, 109.44
7	CH	142.30	7.56, d (15.6)	6.31	165.86, 122.66, 117.19, 109.44
8	CH	117.19	6.31, d (15.6)	7.56	165.86, 142.30, 127.10
9	C	165.86	-		
1′	C	170.42	-		
2′	CH	51.95	5.10, dt (7.7; 3.5)	4.64	170.42, 165.86, 64.18
3′	CH_2	64.18	4.64, m	5.10	170.42, 167.41
1′′	C	109.80	-		
2′′	C	150.72	-		
3′′	CH	116.34	6.63, d (8.9)	7.24	116.74, 109.80
4′′	CH	134.57	7.24, t (9.5)	6.63, 6.60	150.72, 131.09
5′′	CH	116.74	6.60, t (9.0)	7.75, 7.24	116.34, 109.80
6′′	CH	131.09	7.75, d (8.1)	6.60	167.41, 150.72, 134.57
7′′	C	167.41	-		
OCH_3	CH_3	52.97	3.80, s		170.42

[a]Chemical shifts in ppm.

Table S5. Primers sequence

Gene Name	Sequence (5′ → 3′)
ACT1	F: AGCCCAATCCAAAAGAGGTATT<br>R: GCTTGGGTCAACAAAACTGG
ALS3	F: CTAATGCTGCTACTGATAATT<br>R: CCTGAAATTGACATGTAGCA
HWP1	F: CAGCCACTGAAACACCAACT<br>R: CAGAAGTAACAACAACAACACCAG
SAP1	F: TTCATCGCTCTTGCTATTGCTT<br>R: TGACATCAAAGTCTAAAGTGACAAAACC
SAP2	F: TCCTGATGTTAATGTTGATTGTCAAG<br>R: TGGATCATATGTCCCCTTTTGTT
SAP6	F: CCTTTATGAGCACTAGTAGACCAAACG<br>R: TTACGCAAAAGGTAACTTGTATCAAGA
HOG1	F: GACTTGTGGTCTGTGGGTTG<br>R: ACATCAGCAGGAGGTGAGC
ERG11	F: ATTGTTGAAACTGTCATTG<br>R: CCCCTAATAATATACTGATCTG

Table S6. Fractional Inhibitory Concentration Index (FICI) values for the combinations of compounds 1–4 with Fluconazole against *Candida albicans* clinical isolate.

Compound	MIC (µg/mL)	FLC (µg/mL)	MIC alone (compound / FLC)	FICI	Interpretation
1	2.5	2.5	70 / 64	0.075	Synergy
2	2.5	2.5	70 / 64	0.075	Synergy
3	5.0	5.0	200 / 64	0.103	Synergy
4	5.0	2.5	175 / 64	0.068	Synergy
